# Renin-angiotensin-aldosterone system blockers in Bulgarian COVID-19 patients with or without chronic kidney disease

**DOI:** 10.1097/MD.0000000000031988

**Published:** 2022-12-02

**Authors:** Rumen Filev, Lionel Rostaing, Mila Lyubomirova, Boris Bogov, Krassimir Kalinov, Dobrin Svinarov

**Affiliations:** a Department of Nephrology, Internal disease Clinic, University Hospital “Saint Anna,” Sofia, Bulgaria; b Medical University Sofia, Bulgaria; c Nephrology, Hemodialysis, Apheresis and Kidney Transplantation Department, Grenoble University Hospital, Grenoble, France; d Grenoble Alpes University, Grenoble, France; e Head Biometrics Group, Comac-Medical Ltd, Sofia, Bulgaria; f Department of Clinical Laboratory, University Hospital “Alexandrovska,” Sofia, Bulgaria.

**Keywords:** acute kidney injury, chronic kidney disease, COVID-19, ICU admission, mortality, RAAS

## Abstract

When angiotensin-converting enzyme inhibitor/angiotensin receptor blocker-treated patients present with SARS-CoV-2 infection there is a debate to know whether renin-angiotensin-aldosterone (RAAS) blockers should be stopped or not. We conducted a prospective observational study in Bulgarian COVID-19-infected patients with or without chronic kidney disease (CKD) to assess whether maintenance RAAS blocker therapy has an impact on SARS-CoV-2 infection and its complications. We included 120 in-patient COVID-19 subjects, of whom 70 had CKD and 50 had normal renal function. A total of 30% of the patients (total number of 36 patients, 21 females) were receiving RAAS therapy at admission and it was maintained throughout hospitalization. The overall mortality was 19.2% (23 patients); there was no significant difference across the 2 groups (*P*-value = .21), except in RAAS blockers-treated hypertensive patients who had a significantly lower mortality as compared to non-RAAS-blockers-treated hypertensive patients (*P* = .04). Regarding subsequent intensive-care unit admission, there were 50% less patients in the RAAS group (4 out of 36, i.e., 11%) as compared to 19 out of 84 from the non-RAAS group, that is, 22.6% (*P* = .29). Overall, 37 patients developed acute kidney injury (any stage by KDIGO); of them 14 (37.8%) were receiving RAAS blockers. Acute kidney injury was not significantly associated with the use of RAAS blockers (*P*-value = .28). Likewise, both in non-CKD and in CKD patients the use of RAAS blockers did not have an impact on renal function recovery after SARS-CoV-2 infection. Finally, regarding RAAS blockers and the biological parameters outcome only D-dimers were significantly lower at the follow-up as compared to that in non-RAAS blocker treated patients. RAAS blockers benefited patients with hypertension by lowering mortality rate. Other than that, RAAS blocker therapy continuation during SARS-CoV-2 infection in CKD and non-CKD patients had no significant impact upon major outcomes.

## 1. Introduction

The SARS-CoV-2-related coronavirus 2019 (COVID-19) pandemic has killed millions of people worldwide (https://en.wikipedia.org/wiki/Template:COVID-19_pandemic_data). From a medical perspective, the crisis in Bulgaria has not differed from the rest of the world: however, the statistics have revealed disturbing tendencies, that is, the percentage vaccinated in Bulgaria has been 1 of the lowest in Europe and mortality rate has been 1 of the highest in the European Union.^[1]^

SARS-CoV-2 infection disproportionately affects older people with hypertension, diabetes mellitus, chronic kidney disease (CKD), and cardiovascular disease. Patients with these comorbidities are often treated with renin-angiotensin-aldosterone system (RAAS) inhibitors, including angiotensin-converting enzyme inhibitors (ACEIs) and angiotensin receptor blockers (ARBs).^[2–4]^

Antihypertensive drugs, including RAAS inhibitors, have been reported to increase the levels of angiotensin converting enzyme 2, which is a functional receptor for SARS-CoV-2.^[5]^ These observations have prompted concern that the administration of RAAS inhibitors may facilitate SARS-CoV-2 infection and worsen the prognosis. However, a large community-based study has shown that the use of angiotensin-converting enzyme inhibitor (ACEI) or (ARB) was not associated with COVID-19 − confirmed case rate.^[6]^ The same study has also shown that neither ACEI nor ARB use rates were associated with COVID-19 death rates. In addition, a multicenter cohort study including > 1.3 million patients with hypertension from the USA and Spain showed that there was no association between COVID-19 diagnosis and exposure to ACEI/ARB therapy versus calcium-channel blockers/thiazide therapy, as well as no significant difference between RAAS blockers and calcium-channel blockers/thiazides for risk of COVID-19 hospitalization, acute respiratory distress syndrome, acute kidney injury (AKI) or sepsis across all comparisons.^[7]^ Finally, in a meta-analysis, Kaur et al have found that RAAS blockers are not associated with increased mortality in COVID-19 patients.^[8]^

When an ARB or ACEI-treated patient presents with COVID-19, there is a debate to know whether RAAS should be stopped or not. In a randomized controlled trial, Lopes et al have demonstrated that RAAS blocker discontinuation was not associated with beneficial effects for the primary outcome (mean between-group difference in days alive and out of hospital), suggesting that withdrawal of RAAS blockers in hospitalized COVID-19 patients does not alter the short-term prognosis of the disease.^[9]^ In the ACEI-COVID trial, COVID-19 patients on chronic treatment with an ACEI/ARB were randomized to continuation or discontinuation of RAAS blockers for 30 days.^[10]^ The primary endpoint was the maximal Sequential Organ Failure Assessment score within 30 days and was not significantly different between the 2 arms. As a result, RAAS blockers are safe during SARS-CoV-2 infection, and their discontinuation does not appear to alter outcomes. Consequently, several scientific societies have recommended against discontinuation of RAAS blockers in patients with or at risk of SARS-CoV-2 infection.^[11]^ Finally, Smith et al have recently reported on a very large cohort of COVID-19-infected patients that ACEI and ARB exposure have no detrimental effect on hospitalizations and may reduce death among hypertensive patients diagnosed with COVID-19.^[12]^ However, regarding the impact of RAAS blockers in the population of CKD patients having SARS-CoV-2 infection, there are no specific data. This is the reason why we conducted a prospective observational study in Bulgarian COVID-19-infected patients with or without CKD, in which we assessed whether maintenance RAAS blocker therapy has an impact on COVID-19 and its complications.

## 2. Patients and methods

This single-center study was performed at Alexandrovka’s Hospital in Sofia (Bulgaria) between February 1, 2021 and March 31, 2021. We included consecutive patients that were confirmed to have COVID-19 and were admitted into hospital after a positive PCR-test for SARS-CoV-2. All cases were confirmed using the reverse transcription-polymerase chain reaction from combined throat/nose samples.^[13,14]^ Only patients aged > 18 years were included in this study. Patients with a concomitant urinary-tract infection were excluded.

Of the 120 patients enrolled into our study: 70 had a history of CKD (i.e., impaired kidney function with estimated glomerular filtration (eGFR) rate of < 60 mL/minute; however, none had end-stage renal disease. The other 50 patients had no history of kidney disease and had normal levels of serum creatinine (i.e., females 44–80 µmol/L; males 62–106 µmol/L; eGFR CKD-EPI 2021 of > 60 mL/minute/1.73 m^2^). The control group also included 5 patients that had undergone renal transplantation (eGFR of > 60 mL/minute). None of the patients were vaccinated.

Data on patients’ gender, age, comorbidities, and laboratory results from blood taken at admission into the emergency room, were collected. A follow up was done at 7 to 10 days after admission.

Comorbidities included hypertension, obesity (i.e., body mass index of ≥ 30 kg/m^2^), diabetes mellitus, vascular disease, and CKD, which had to be already diagnosed along with its medical history. Kidney function was estimated by CKD-EPI 2021.

The study was conducted according to the guidelines of the Declaration of Helsinki and approved by the ethical committee KENIMUS of the Medical University of Sofia, Bulgaria, with Protocol No. 12/31.05.2022. All data are available upon request from the corresponding author.

## 3. Statistical analysis

### 3.1. Methods for data description

1. Categorical parameters were analyzed according to absolute and relative (percentage) frequencies.
2. Continuous parameters were analyzed according to arithmetic mean, standard deviation, and median, minimum, and maximum values.

### 3.2. Methods for testing post hoc hypotheses

1. To compare continuous parameters in 2 related (paired) groups, Student’s paired sample *t* test was applied.
2. The exact chi-squared test was used to compare non-metric parameters from the 2 independent (unrelated) groups (angiotensin receptor blocker/angiotensin converting enzyme inhibitor [ACEi/ARB] user vs non-users).
3. Continuous variables for independent groups (ACEi/ARB user vs non-user) were analyzed using a general linear model with the baseline value as the covariate and the group as the factor.

For decision making, a significance level of 5% was used. SAS® package ver. 9.4 was used for the calculations and graphical presentations.

Logistic regression was used to model the relation between output as a dependent variable and the main parameters. A model is presented for mortality. In addition, odds ratios are shown. For decision making, a significance level of 5% was used.

The SAS® package ver. 9.4 was used for the calculations and the graphical presentations.

## 4. Results

Of the 120 confirmed COVID-19 patients, 70 (58.3%) had a history of CKD. Thirty percent of the patients (total number of 36 patients) were receiving therapy with ACEi or ARB: that is, 21 females (58.3%) and 15 males (41.7%). The other 84 patients did not receive ACEi or ARB.

All patients receiving ACEi/ARB had been receiving this therapy before becoming infected with SARS-CoV-2; none were prescribed with ACEi/ARB during the SARS-CoV-2 infection.

In the non-ACEi/ARB group, some patients had hypertension: that is, 36 (42.9%). They were taking different classes of anti-hypertension drugs, and all had been prescribed well before COVID-19.

In Table 1, patients are compared for their demographic factors and comorbidities. Those not receiving ACEi/ARB therapy had significant differences in arterial hypertension, obesity, CKD, and cardiovascular disease compared to those receiving ACEi/ARB. Comorbidities such as autoimmune diseases, COPD/asthma and hypothyroidism were not taken in account, which can be a limitation to this analysis.

**Table 1:** Demographics and comorbidities at baseline in the 2 groups.

**Demographics and Comorbidities**	**No therapy with ACEI/ARB**	**Therapy with ACEI/ARB**	***P*-value**
Number of patients	84	36	
Age	65.5 (19–88)	65.5 (42–80)	.09
Gender	Female—39 (46.4%)	Female—21 (58.3%)	.23
	Male—45 (53.6%)	Male—15 (41.7%)	
Race	100% Caucasian	100% Caucasian	
Obesity	No—76 (90.5%)	No—27 (75.0%)	.02
	Yes—8 (9.5%)	Yes—9 (25.0%)	
Cardiovascular diseases	No—59 (70.2%)	No—18 (50.0%)	.03
	Yes—25 (29.8%)	Yes—18 (50.0%)	
Diabetes	No—58 (69.0%)	No—21 (58.3%)	0.25
	Yes—26 (31.0%)	Yes—15 (41.7%)	
Hypertension	No—36 (42.9%)	No—5 (13.9%)	.002
	Yes—48 (57.1%)	Yes—31 (86.1%)	
CKD	No—40 (47.6%)	No—10 (27.8%)	.04
	Yes—44 (52.4%)	Yes—26 (72.2%)	

ACEI = angiotensin-converting enzyme inhibitor, ARB = angiotensin receptor blocker, CKD = chronic kidney disease.

Table 2 shows comparisons between the 2 groups and its purpose is to determine whether ACEi/ARB therapy had an impact on mortality on the first place and secondly if it was associated with lower rate of AKI. The mortality rate within our complete cohort (120 patients) was 19.2% (23 patients). Four of the patients that died were from the ACEi/ARB group and the rest of the 15 patients were from the control group. There was no significant difference across the 2 groups with regards to the mortality rate (*P*-value = .21).

**Table 2:** Influence of ACEI or ARB use on Death Rate and AKI.

Parameter		No therapy with ACEI/ARB	Therapy with ACEI/ARB	*P*-value	RR (95% CI)
Death					
No	n%	65 (77.38%)	32 (88.89%)	.21	0.49 (0.18; 1.34)
Yes	n%	19 (22.62%)	4 (11.11%)		
Death (Hypertension only)					
No	n%	31 (64.58%)	27 (87.10%)	.04	0.36 (0.14; 0.98)
Yes	n%	17 (35.42%)	4 (12.90%)		
Death (Diabetes only)					
No	n%	18 (69.23%)	14 (93.33%)	.12	0.22 (0.03; 1.57)
Yes	n%	8 (30.77%)	1 (6.67%)		
Death (Diabetes and Hypertension)					
No	n%	17 (68.00%)	14 (93.33%)	.12	0.21 (0.03; 1.51)
Yes	n%	8 (32.00%)	1 (6.67%)		
AKI					
No	n%	61 (72.62%)	22 (61.11%)	.28	0.84 (0.63; 1.13)
Yes	n%	23 (27.38%)	14 (38.89%)		

ACEI/ARB = angiotensin-converting enzyme inhibitor/angiotensin receptor blocker, AKI = acute kidney injury, RR = risk ratio, CI = confidence interval.

Following admission (day 0), 23 patients subsequently required intensive care unit (ICU) admission: all were ventilated. Of these there 4 from the ACEi/ARB group (11%) and 19 from the non ACEi/ARB group (22.6%), that is, OR = 0.4276 (95% confidence interval: [0.0983, 1.4467]; *P*-value = .29).

Also, we did the same analyses in subgroups of patients having hypertension and/or diabetes, (i.e., comorbidities that had been diagnosed before the SARS-CoV-2 infection). Statistically, fewer hypertensive patients receiving RAAS blocker therapy had died (*P*-value = .04) as compared to the patients who had hypertension and who were not treated by RAAS blockers. There was no statistical difference for patients with either diabetes (*P*-values = .12) or hypertension and diabetes (*P*-values = .12) and taking or not taking RAAS blockers, so we found no benefit of the ACEi/ARB therapy for that group of patients.

The same analysis was performed for the 2 groups to see if the patients who had developed AKI whilst being treated for COVID-19 had any impact by the ACEi/ABR therapy. In total, there were 37 patients with AKI (any stage by KDIGO) across the 120 patients. Of them, 14 (37.8%) were receiving ACEi/ARB. The comparison between the groups of patients taking ACEi/ARB and the non-ACEI/ARB group didn’t show any impact—positive nor negative for those of the patients who had developed AKI (*P*-values = .28).

To assess if the usage of ACEi/ARB affected and/or improved the laboratory parameters, we compared the 2 groups between baseline and follow-up for the parameters of eGFR (CKD-EPI 2021), D-dimer, albumin, and total protein (Table 3). The table shows that there was no significance between the different variables except for D-dimer, which was significantly lower at the follow up for patients that received ACEi/ARB on a regular basis (*P*-values = .01).

**Table 3:** Laboratory parameters for ACEi/ARB versus Non-ACEi/ARB patients.

Parameter	Statistics	Baseline		Follow-up (7–10 days)		Change		*P*-value
		ACEI/ARB	No ACEI/ARB	ACEI/ARB	No ACEI/ARB	ACEI/ARB	No ACEI/ARB	
*Number of* *patients*	N	36	84	36	84	36	84	
*eGFR*	Mean (SD)	58.8 (27.56)	65.0 (31.34)	73.4 (27.05)	72.6 (33.70)	14.6 (15.71)	7.6 (20.18)	.10
*D-Dimer*	Medium (Ranges)	0.8 (0.3–5.5)	0.6 (0.3–10.7)	0.8 (0.3–4.0)	0.5 (0.3–10.7)	−0.3 (−2.1–0.4)	0.0 (−4.4–6.1)	.01
*Albumin*	Medium (Ranges)	63.0 (46.0–71.0)	65.0 (50.0–89.0)	59.0 (46.0–70.0)	61.5 (42.0–72.0)	−2.0 (−14.0–18.0)	0.0 (−24.0–6.0)	.60
*Protein*	Medium (Ranges)	35.0 (28.0–44.0)	37.0 (25.0–48.0)	34.0 (25.0–41.0)	34.0 (25.0–48.0)	−1.5 (−9.0–13.0)	0.0 (−16.0–8.0)	.43

ACEI/ARB = angiotensin-converting enzyme inhibitor/angiotensin receptor blocker, eGFR = estimated glomerular filtration rate, DF = degrees of freedom, SD = standard deviation.

*From ANOVA model with measurement at Visit2 as dependent, measurement at Baseline as covariate, and RAAS as fixed effect factor.

Estimates are based on the number of subjects with non-missing data.

**Figure 1.**
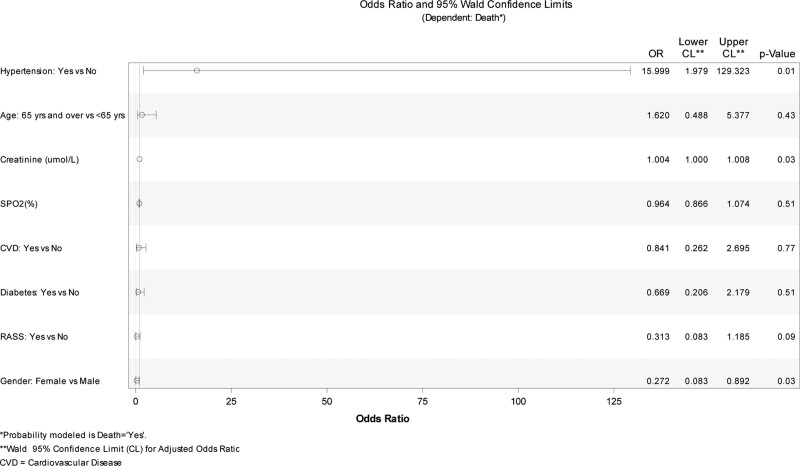
Odds ratio (Independent predictors for COVID-19 related death).

We then analyzed the risk factors that may have led to death in patients with RAAS therapy and those without RAAS therapy. Table 4 shows logistic regression results for the mortality risk factors. Statistically significant independent risk factors for mortality were creatinine level at admission over the baseline values (male over 106 µmol/L and for females over 80 µmol/L) [OR = 1.004 (1.000–1.008); *P* = .03] and having hypertension [OR = 15.99 (1.97–129.32); *P* = .001], whereas female gender was protective [OR = 0.272 (0.083–0.892); *P* = .03]. These results were confirmed by assessing the odds ratio: the results can be seen in Figure 1.

**Table 4:** Logistic Regression Model: Odds Ratios for death prediction.

Effect	Odds Ratio	Lower CL**	Upper CL**	*P*-Value
Hypertension: Yes versus No	15.999	1.979	129.323	.001
Age: 65 yrs and over versus < 65 yrs	1.620	0.488	5.377	.43
Creatinine (umol/L)	1.004	1.000	1.008	.03
SPO2 (%)	0.964	0.866	1.074	.51
CVD: Yes versus No	0.841	0.262	2.695	0.77
Diabetes: Yes versus No	0.669	0.206	2.179	.50
RAAS: Yes versus No	0.313	0.083	1.185	.08
Gender: Female versus Male	0.272	0.083	0.892	.03

CVD = cardiovascular diseases, RAAS = renin-angiotensin-aldosterone system, SpO2 = peripheral oxygen saturation, yrs = years.

Because of the importance of using ACEi/ARB to protect kidney function a statistical comparison was made to compare the baseline kidney function (i.e., at admission) with serum creatinine levels before the SARS-CoV-2 infection (at least a month before) and then the results were compared with the 7 to 10-day follow-up. Factors compared were creatinine level (normal range for females 44–80 µmol/L, males 62–106 µmol/L) and eGFR was calculated using the CKD-EPI 2021 formula. The results showed that eGFR (*P*-value = .66) and serum creatinine (*P*-value = .17) were not statistically different despite an initial decline in renal function in the course of SARS-CoV-2 infection, that is, there was after the acute phase of the infection a subsequent improvement in renal function and recovery of serum creatinine and GFR levels to values that were similar to those the patients had before the SARS-CoV-2 infection.

## 5. Discussion

In this real-life study, we found that COVID-19-infected patients having ARB or ACEI therapy at admission in the emergency room had the same outcome as those not having these therapies (i.e., mortality rate, ICU admission, or rate of the AKI, regardless having or not having at baseline CKD). In addition, the independent predictive factors for COVID-19-related death were prior history of hypertension and creatinine at admission, whereas the female gender was significantly protective. Moreover, the RAAS-blocker-treated hypertensive patients had a significantly lower mortality rate. This analysis is the first of its kind and has never been done in the past for such group of patients in Bulgaria.

It is well known that COVID-19-related mortality is associated with gender, (i.e., male COVID-19 patients have a significantly higher mortality rate than female COVID-19 patients).^[15,16]^ But in our group of patients we did not find any difference for the patients on ACEi/ARB therapy when compared to the control group in the statistical analysis by gender. Women appear to be less prone to severe forms of the disease and their mortality is lower than for men. The role of female hormones in the modulation of inflammation may be the reason behind this gender difference.^[17]^ Indeed, in our study we found that the female gender was significantly associated with less COVID-19-related mortality.

At the beginning of the pandemic there was a big issue regarding being on RAAS inhibitor treatment at the time of SARS-CoV-2 infection because RAAS inhibitors increase the levels of angiotensin converting enzyme 2, which is a functional receptor for SARS-CoV-2.^[5]^ However, since that time there have been randomized controlled trials in COVID-19 patients on RAAS inhibitors at hospital admission in whom RAAS inhibitors were either maintained or discontinued.^[9,10]^ These studies have shown that the outcomes (ICU admission, mortality) were not affecting whatsoever the RAAS inhibitor strategy at the time of SARS-CoV-2 infection. Indeed, in all our COVID-19 patients we maintained RAAS inhibitor therapy when already present at admission. Finally, 2 meta-analyses were reassuring: Kaur et al found that RAAS blockers are not associated with increased mortality in COVID-19 patients and should be continued in hypertensives^[8]^ and more recently Singh et al reported that the use of ACEIs/ARBs did not significantly influence either mortality (OR = 1.16 95% CI: 0.94–1.44, *P* = .15, *I*^2^ = 93.2%) or severity (OR = 1.18, 95% CI: 0.94–1.48, *P* = .15, *I*^2^ = 91.1%) in comparison to not being on ACEIs/ARBs in COVID-19 positive patients.^[18]^

Bae et al studied a cohort of 1374 hypertensive patients with COVID-19, of which 1076 (78.3%) and 298 (21.7%) were users and never-users of RAAS inhibitors, respectively. The primary outcome was the composite of intensive care unit admission, invasive mechanical ventilation, continuous renal replacement therapy (CRRT), extracorporeal membrane oxygenation (ECMO), and death from COVID-19. They found that the RAAS inhibitor users were not associated with the risk of the primary outcome; conversely, the risk of ICU admission was significantly lower in the users than the never-users (aOR, 0.44; 95% CI: 0.24 to 0.84), particularly in patients who did not require invasive mechanical ventilation.^[19]^ Indeed, we found that as compared to non-RAAS inhibitor-treated COVID-19 patients RAAS inhibitor-treated COVID-19 patients were 50% less likely to be subsequently admitted in ICU (i.e., 11% vs 22.6%) even though the difference was not statistically significant.

In COVID-19 infected patients we observed that developing AKI after hospital admission was an independent risk factor for mortality.^[20]^ So, from a nephrological perspective it is really important to investigate whether RAAS blocker-treated COVID-19 patients have any benefit or harm from the treatment with that class of antihypertensive drugs. At the early stages of the pandemic with SARS-CoV-2, Oussalah et al found in a cohort of 149 COVID-19 patients of whom 30% (44/149) were treated with ACEI/ARB that ACEI/ARB use was independently associated with AKI stage ≥ 1 (OR, 3.28, 95% CI: 2.17, *I*^2^ = 4.94).^[21]^ In a systematic review and meta-analysis in 2021, Lee et al found that RAAS blockade use is significantly associated with the incident AKI in hospitalized COVID-19 patients.^[22]^

Our study is almost the same size as that from Oussalah et al.^[21]^ However, we found that having ACEi/ARB therapy before admission and being maintained on that therapy after admission has no impact on the outcome of the SARS-CoV-2 infection, including no increased risk for AKI.

In a large cohort of 2726 COVID-19 patients from 178 US hospitals, Banwait et al have shown that taking ACEIs and ARBs prior to admission for COVID-19 was independently associated with decreased need for mechanical ventilation and in-hospital mortality.^[23]^ In our study we did not observe that COVID-19 patients who were on ACEi/ARB therapy before admission were protected from subsequent referral to the ICU.

In a meta-analysis including a total of 1808 patients with hypertension diagnosed with COVID-19 in 6 studies, Xue et al found that being treated with ACEI/ARB drugs had positive effect on reducing D-dimer and the number of people with fever; however, it had no significant effect on other laboratory tests.^[24]^ Indeed, we also observed in our study that COVID-19 patients on RAAS blockade therapy before and during hospitalization had reduced D-Dimer. However, we did not observe that RAAS blockade therapy had any significant effect on other laboratory tests in patients with COVID-19.

In conclusion, in this real-life single center study from Bulgaria we found that COVID-19 patients with or without CKD who were on RAAS blockade at admission and who were maintained on that therapy during hospitalization had the same outcomes in terms of mortality, ICU admission and AKI rate as those who were not on RAAS blockade. In addition, the RAAS-blocker-treated hypertensive patients had a significantly lower mortality rate. This means that the therapy with ACEi/ARB should not be halted in COVID-19, because of its beneficial effect for controlling the blood pressure and maybe on mortality rate.

## Author contributions

**Conceptualization:** Rumen Filev, Mila Lyubomirova, Boris Bogov, Dobrin Svinarov.

**Data curation:** Rumen Filev.

**Formal analysis:** Rumen Filev, Krassimir Kalinov.

**Funding acquisition:** Rumen Filev, Lionel Rostaing.

**Investigation:** Rumen Filev.

**Methodology:** Rumen Filev, Krassimir Kalinov, Dobrin Svinarov.

**Project administration:** Mila Lyubomirova, Boris Bogov.

**Supervision:** Mila Lyubomirova, Boris Bogov.

**Writing – original draft:** Rumen Filev.

**Writing – review & editing:** Lionel Rostaing.
